# Symptoms of depression, anxiety and stress in patients with chronic otitis media

**DOI:** 10.1371/journal.pone.0270793

**Published:** 2022-07-01

**Authors:** Ana D. Jotic, Ana M. Opankovic, Zorana Z. Radin, Ljiljana Cvorovic, Katarina R. Savic Vujovic, Sanja B. Krejovic-Trivic, Bojana M. Bukurov, Biljana R. Milicic, Jasmina D. Stojanovic

**Affiliations:** 1 Clinic for Otorhinolaryngology and Maxillofacial Surgery, Clinical Center of Serbia, Belgrade, Serbia; 2 Medical Faculty, University of Belgrade, Belgrade, Serbia; 3 Clinic for Psychiatry, Clinical Center of Serbia, Belgrade, Serbia; 4 Ear, Nose and Throat Clinic, Clinical Hospital Center Zvezdara, Belgrade, Serbia; 5 Department of Pharmacology, Clinical Pharmacology and Toxicology, Faculty of Medicine, University of Belgrade, Belgrade, Serbia; 6 Faculty of Dental Medicine, University of Belgrade, Belgrade, Serbia; 7 Clinic for Otorhinolaryngology, Clinical Hospital Center Kragujevac, Kragujevac, Serbia; 8 Faculty of Medical Sciences, University of Kragujevac, Kragujevac, Serbia; Universidade Federal de Sao Paulo/Escola Paulista de Medicina (Unifesp/epm), BRAZIL

## Abstract

**Purpose:**

Persistent symptoms of chronic otitis media cause limitations in daily routine and social interactions, influencing significantly patients’ quality of life and mental health. The purpose of the study was to assess the intensity depression, anxiety and stress symptoms in patients with chronic otitis media and to examine if patient demographic data, characteristics and reported symptoms of otitis influence reported depression, anxiety and stress symptoms.

**Material and methods:**

The study included 316 adult patients diagnosed with unilateral or bilateral chronic otitis media with or without cholesteatoma. Patients underwent a complete otological, audiological and radiological assessment. Chronic otitis media questionnaire 12 (COMQ-12) was used to assess the impact of COM and Depression Anxiety Stress Scale 21 (DASS-21) was used for depression, anxiety and stress assessment.

**Results:**

Some level of anxiety and stress were detected in 70.57% 49.37% of the patients, respectively. 13.29% of the patients had scores indicating depression disorder. The mean value of the COMQ-12 questionnaire for this group of patients was 26.24 (SD±11.47) More intense symptoms of COM were significantly associated (p<0.05) with higher scores on DASS-21 subscales. Multivariate logistic regression analysis indicated that significant positive predictors of higher anxiety scores were pure tone average (PTA) on better and worse hearing ear (p<0.05). Drainage from the ear, hearing problems at home and tinnitus were significant positive predictors of a higher DASS-depression score. (p<0.05)

**Conclusion:**

The study confirmed positive correlation between reported level of anxiety, depression and stress, severity of COM symptoms and quality of life. Level of hearing on the better and worse hearing ear were significant positive predictors of anxiety and stress in patients with COM.

## Introduction

Chronic otitis media (COM) is a worldwide public health problem that affects from 65 to 330 million people worldwide, where about 50% of presenting individuals have significant degree of hearing impairment. It was estimated that COM and its complications are responsible for 21–28 000 deaths annually [1].

The disease involves prolonged chronic inflammation of the middle ear and mastoid cavity, which can lead to damage of the tympanic membrane, ossicular chain and surrounding bone [2, 3]. Recurrent ear discharge or otorrhea through a tympanic membrane perforation is present in active forms of the disease. COM can be classified as mucosal or squamous form, with or without cholesteatoma. Compared to the mucosal form of COM, this form is more destructive and more frequently results in ossicular chain destruction, bone destruction and the occurrence of complications (facial nerve paralysis, labyrinthitis, mastoiditis, meningitis, epidural, subdural and brain abscess formation). The most common sequelae of the disease involve hearing loss, tinnitus and vertigo. Treatment of COM involves repeated use of antimicrobial agents, but ultimately requires surgical management to avoid further advancement of the disease and possible life-threating complications [4].

Persistent symptoms of COM cause limitations in daily routine and social interactions, markedly influencing a patient’s quality of life (QOL) and mental health [5]. Recently, there has been growing interest in and recognition of the importance of psychological disorders, particularly anxiety and depression, in patients with COM [6–9]. Patients with anxiety and depressive disorders exhibit lower adherence to treatment, fewer positive health behaviors, higher risk of adverse outcomes and increased medical costs [7]. Anxiety and depression symptoms can have profound effects regarding the control of COM, post-treatment results, the patient’s QOL and general health. It is surprising that only a few published studies up to date examined the actual level of anxiety or depression symptoms in COM patients and that reference to this disorders is scarce in general COM literature [7–9].

The recently developed COM questionnaire 12 (COMQ-12) provides insight into overall disease burden from a patient’s rather than clinician’s perspective. This proved to be extremely important, since clinical, radiologic and audiologic findings are poor predictors of the quality of life in COM patients [5]. The improved understanding of a patient’s experience and prioritization of their symptoms could be used to assess the effect of COM on their mental health.

The aim of the present study was to assess the occurrence and intensity of depression, anxiety and stress symptoms in patients in patients with COM with and without cholesteatoma using two specific questionnaires. The secondary goals were to examine if the demographic patient data, clinical characteristics, audiological findings, intensity of specific reported otitis symptoms and the QOL influence reported depression, anxiety and stress symptoms.

## Material and methods

### Study design and population

A cross-sectional study was conducted on 316 patients diagnosed with unilateral or bilateral COM with or without cholesteatoma. The study included adult patients (≥18 years) of both sex who were diagnosed at the Clinic for Otorhinolaryngology and Maxillofacial Surgery, Clinical Centre of Serbia, from October 2017 to October 2019. Exclusion criteria were as follows: previously surgically treated COM with cholesteatoma (squamous) or without cholesteatoma (mucosal), existence of any other otorhinolaryngology disease and previous diagnosis of anxiety, depression or any other psychiatric diseases. The study was approved by the Ethics Committee of the Clinical Center of Serbia (36/V-12/17).

In addition, the questionnaires were administered to a normative reference control group of 110 chosen among volunteers (medical students, hospital staff, and families of the authors). Every subject was examined by the authors (two otorhinolaringologists and a psychiatrist) prior to filling out the questionnaires. Subjects who had who had pathological findings on their otorhinolaryngological exam, who had history of COM, previous ear surgery or any other otorhinolaryngology disease were excluded from the study. Also, subjects with previous diagnosis of anxiety, depression, with any other psychiatric diseases or psychiatric medication were excluded from the study.

### Clinical examinations and symptoms assessment

All patients underwent complete otorhinolaryngology examination, otomicroscopy, pure-tone audiometry of both ears with air- and bone-conduction hearing levels and computerized tomography (CT) of the temporal bone. Normative reference volunteer group underwent otorhinolaryngology examination, otomicroscopy, pure-tone audiometry. Audiometry was performed with Interacoustics clinical audiometer AC 40 (Interacoustics, Middelfart, Denmark). For determining air-conduction thresholds, the supra-aural transducer was used. Contralateral masking was applied if the obtained bone-conduction (non-test ear) and air-conduction (test-ear) difference for the frequency being tested was greater than 40 dB, and always when determining bone conduction thresholds [10] To assess hearing bilaterally, the pure tone average (PTA) was calculated as the average of hearing threshold levels (in dB) at specified frequencies of 500, 1000, 2000 and 4000 Hz for the better and worse hearing ears. Other data aquired from the patients and their medical history included age, sex, marriage/partnership status, level of education (primary school, secondary school, post-secondary education, higher education), and the duration of the disease (duration of up to 1 year, from 1 to 3 years, from 3 to 5 years, duration over 5 years).

The COMQ-12 [11] was used to assess the impact of the disease. The questionnaire consists of twelve questions related to the symptoms severity (discharge or drainage from the ear, having a ‘‘smelly ear”, hearing problems at home, hearing problems when talking to people in groups or when there are noisy surroundings, discomfort in and/or around the ear, dizziness or feeling ‘‘off balance”, tinnitus); lifestyle and work impact (perform normal daily activities at home, taking showers and baths); health service impact (general practice doctor visits, medication use) and the general impact of the disease on the patient’s life. The severity of each symptom was assessed on a scale from 0 to 5. Higher scores indicated a poorer QOL in patients with COM, with maximum score of 40.

The DASS-21 [12, 13] was used to assess depression, anxiety and stress symptoms. The DASS-21 consists of three self-report scales with seven items designed to measure the negative emotional status of depression, anxiety and stress. The depression scale evaluates dysphoria, hopelessness, devaluation of life, self-depression, lack of interest/ involvement, anhedonia and inertia. The anxiety scale assesses autonomic arousal, skeletal muscle effects, situational anxiety and the subjective experience of anxiety. The stress scale is sensitive to the levels of chronic non-specific arousal. It assesses difficulty in relaxing, nervous arousal, and being easily upset/agitated, irritable/overactive and impatient. Recommended cut-off scores for the severity of depression are mild (between 5 and 6), moderate (7–10), severe (11–13) and extremely severe (over 14). Depression scores from 0 to 4 were considered normal. Recommended cut-off scores for the severity of anxiety were mild (4 and 5), moderate (6–7), severe (8–9) and extremely severe (over 10). Anxiety scores from 0 to 6 were considered normal. Stress was rated as mild (when score was between 8 and 9), moderate (10–12), severe (13–16) and extremely severe (over 17). Stress scores from 0 to 7 were considered normal. The questionnaires were self-reported, and all patients were previously given information and an explanation on how to answer them.

### Sample size and statistical analysis

Statistical analysis was performed using the IBP SPSS Statistics v28 (Statistical Package for Social Sciences, SPSS Inc., Chicago, Illinois). If the significance level α = 0.05 (5%) and the statistical power of the test 1-β = 0.8 (80%), the required sample size of 316 subjects with COM, based on data from previously published studies with the frequency of occurrence anxiety and depression 28% in the population. Descriptive statistics were calculated for demographic characteristics and were presented as frequencies and proportions and with mean, median and standard deviation. Man-Whitney and Kruskal-Wallis were used to compare COMQ-12 items and DASS-21 scores with demographic patient data, clinical characteristics and audiological findings. Pearson and Spearman’s Rho Correlation was used to establish the correlation between the intensity of reported COM symptoms in COMQ-12 and DASS-21 item scores. For statistical analysis, univariable and multivariable linear regression methods were used to determine the predictors of the DASS-21 item scores. All test variables with a statistical significance of p<0.05 in the univariable model were included in the multivariable model. Statistical significance was considered at p<0.05.

## Results

This cross-sectional study included 316 patients diagnosed with unilateral or bilateral COM with or without cholesteatoma. The average age of the patients was 48.4 years (SD±16), with a similar distribution of sex (49.7% males vs. 50.3% females). Patients with cholesteatoma were more numerous (57.9%). The calculated PTA for the worse hearing ear was 58.43 (SD±15.12), and 29.97 (SD±14) for the better hearing ear. Mean values of DASS-21 items were 2.31 (SD±2.8) for depression, 4.82 (SD±3.31) for anxiety and 5.9(SD±3.49) for stress. The mean value of the COMQ-12 questionnaire was 26.24 (SD±11.47) (Table 1). Based on DASS-21 scores, 42 patients (13.29%) screened positive for some form of depression, from mild to severe; 223 patients (70.57%) had positive scores for anxiety, varying from mild to extremely severe, and 156 patients (49.37%) had positive scores for stress, varying from mild to severe. Co-occurring anxiety disorder was noted in 36 (85.71%) of the patients with depression disorder. Comparing to normative reference volunteer group, there were no significant differences in age, sex distribution, level of education and partnership status between the groups. There were however, significant differences between groups in values depression, anxiety and stress subscales of the DASS-21 questionnaire and the total score of COMQ-12 questionnaire (p<0.001) (Table 1). Maximum scores for all three DASS-21 subscales in the control group in were within the normal range.

**Table 1:** Characteristics of the patients with COM and control group volunteers included in the study.

Characteristic	COM patients	Controls	*P*
**Sex n(%)**			0.104
Male	157 (49.7)	63 (57.3)	
Female	159 (50.3)	47 (42.7)	
**COM type n(%)**			
With cholesteatoma	183 (57.9)	n/a	
Without cholesteatoma	133 (42.1)	n/a	
**Duration of the disease n(%)**			
Up to 1 year	42 (13.3)	n/a	
1 to 3 years	108 (34.2)	n/a	
3 to 5 years	64 (20.3)	n/a	
Over 5 years	85 (26.9)	n/a	
*Missing*	17 (5.4)	n/a	
**Level of education n(%)**			0.223
Primary school	28 (8.9)	15 (13.6)	
Secondary school	76 (24.1)	27 (24.5)	
Post-secondary education	88 (27.8)	34 (30.9)	
High education	110 (34.8)	34 (30.9)	
Missing	14 (4.4)		
**In a marriage/partnership n(%)**			0.297
Yes	197(62.3)	31 (28.2)	
No	91(28.8)	79 (71.8)	
Missing	28 (8.9)		
**PTA air-conduction (mean ±SD, dB)**			<0.001_a_
Worse ear	58.43± 15.12	19.6±4.05	
Better ear	29.97± 14	18.7±3.98	
**PTA bone-conduction (mean ±SD, dB)**			<0.001^a^
Worse ear	20.21±6.13	14.41±4.26	
Better ear	15.48±3.93	14.1±4.01	
**DASS-21 (mean ±SD)**			<0.001^a^
Depression scale	2.31±2.8	0.85±1.07	
Anxiety scale	4.82±3.31	1.2±1.21	
Stress scale	5.9±3.49	1.7±1.6	
**COMQ-12 (mean ±SD)**	26.24±11.47	1.28±1.41	<0.001^a^

PTA–Pure tone average, DASS-21- Depression Anxiety Stress Scale 21, COMQ-12- Chronic otitis media questionnaire 12, dB- decibels

^a^ p value <0.05

Significant differences in DASS-21 depression, anxiety and stress subscale scores and in COMQ-12 total score were noted between different levels of PTA for the better and worse ears (Kruskal-Wallace, p<0.01), but not between other analyzed demographic characteristics (Table 2).

**Table 2:** DASS-21 subscale scores and COMQ-12 scores according to patient’s characteristics.

	DASS–Depression (mean ±SD)	*p*	DASS- Anxiety (mean ±SD)	*p*	DASS-Stress (mean ±SD)	*p*	COMQ-12 (mean ±SD)	*p*
**Sex**								
Male	2.11±2.59	0.513	5±3.42	0.224	5.4±3.39	0.152	26.11±11.23	0.942
Female	2.33±2.88		5.31±3.32		5.96±3.5		26.36±11.75	
**COM type**								
Without cholesteatoma	2.34±2.93	0.952	5.05±3.46	0.149	5.72±3.45	0.905	25.31±11.7	0.111
With cholesteatoma	2.06±2.44		5.3±3.25		5.63±3.47		27.51±11.1	
**Duration of the disease**								
<1y	2.4±2.41	0.458	5.45±3.6	0.707	5.88±3.77	0.923	26.54±10.63	0.828
1-3y	2.37±2.8		4.99±3.4		5.56±3.59		25.6±11.89	
3-5y	2.05±2.8		5.19±3.65		5.67±3.22		26.4±11.86	
>5y	2.15±2.82		5.35±3.01		5.84±3.22		27.5±10.5	
**Level of education**								
PS	2.18±2.96	0.798	4.96±3.65	0.359	5.64±2.83	0.506	24.21±10.27	0.534
SS	2.43±2.89		5.28±3.32		6.29±3.57		26.9±11.66	
PSS	2.17±2.95		4.57±3.16		5.3±3.24		25.11±11.52	
HE	2.12±2.52		5.45±3.47		5.55±3.65		26.72±11.46	
**In a marriage/ partnership**								
Yes	2.13±2.84	0.126	5±3.29	0.533	5.76±3.43	0.945	25.82±11.35	0.862
No	2.29±2.26		5.44±3.57		5.65±3.53		25.95±11.61	
**Worse hearing ear PTA (dB)**								
Mild	4.37±4.84	0.001^a^	3.93±5	0.000^a^	7.31±4.95	0.000^a^	18±10.94	0.000^a^
Moderate	3.57±4.85		7.41±5.88		9.75±6.52		22.24±10.4	
Moderately severe	4.96±5.51		11.09±5.22		12.36±6.27		28.51±10	
Severe	6.31±7.26		14.05±6.32		16.96±6.34		33.67±10.19	
Profound	4±4.35		17±5.79		17.81±5.45		38.63±5.8	
**Better hearing ear PTA (dB)**								
Normal to slight	1.75±2.65	0.000^a^	3.61±2.95	0.000^a^	4.26±03	0.000^a^	20.97±10.47	0.000^a^
Mild	2.53±2.55		5.68±2.86		5.85±3		29.58±9.93	
Moderate	2.44±2.45		7.4±2.67		7.87±2.97		32.65±9.73	
Moderately severe	5.83±4.75		8.5±5.65		9.67±4.92		34.67±14.34	
Severe	6.33±5.51		7±5.56		8.33±4.72		32±5.29	

PS-primary school, SS-secondary school, PSS-post secondary education, HE- Higher education. PTA-pure tone average, dB-decibels

^a^ p value <0.05

A highly significant positive correlation was found between DASS-21 depression, anxiety and stress subscale scores and COMQ-12 total score (Pearson correlation, p≤0.001). The scores for the majority of items in COMQ-12 also had a significant positive correlation for DASS-21 subscales for depression, anxiety and stress (Spearman’s Rho, p<0.05) (Table 3).

**Table 3:** Correlation between DASS subscale scores and COMQ-12 scores.

	DASS-Depression		DASS- Anxiety		DASS- Stress	
COMQ-12	Correlation Coefficient (N = 316)	p	Correlation Coefficient (N = 316)	p	Correlation Coefficient (N = 316)	p
**Symptom severity**	0.28	<0.001^a^	0.68	<0.001^a^	0.57	<0.001^a^
Discharge from the ear	0.21	<0.001^a^	0.53	<0.001^a^	0.44	<0.001^a^
‘‘Smelly ear”	0.12	0.034^a^	0.37	<0.001^a^	0.32	<0.001a
Hearing at home	0.23	<0.001^a^	0.59	<0.001^a^	0.43	<0.001^a^
Hearing in groups or in noise	0.18	0.001^a^	0.57	<0.001^a^	0.44	<0.001^a^
Discomfort in the ear,	0.17	0.002^a^	0.52	<0.001^a^	0.43	<0.001^a^
Dizziness	0.15	0.007^a^	0.4	<0.001^a^	0.33	<0.001^a^
Tinnitus	0.14	0.01^a^	0.33	<0.001^a^	0.29	<0.001^a^
**Life and work impact**	0.23	<0.001^a^	0.43	<0.001^a^	0.39	<0.001^a^
Daily activities	0.17	0.003^a^	0.33	<0.001^a^	0.3	<0.001^a^
Taking showers and baths	0.2	<0.001^a^	0.46	<0.001^a^	0.38	<0.001^a^
**Health Service impact**	0.16	0.005^a^	0.44	<0.001^a^	0.35	<0.001^a^
GP visits	0.13	0.025^a^	0.46	<0.001^a^	0.37	<0.001^a^
Medication use	0.13	0.018^a^	0.37	<0.001^a^	0.27	<0.001^a^
**General impact**	0.16	<0.001^a^	0.57	<0.001^a^	0.53	<0.001^a^
**COMQ-12 total**	0.28	<0.001^a^	0.66	<0.001^a^	0.56	<0.001^a^

DASS-21- Depression Anxiety Stress Scale 21, COMQ-12- Chronic otitis media questionnaire 12, GP-General practice

^a^ p value <0.05

According to univariable linear regression, the PTA for the better and worse hearing ear, all items on COMQ-12 questionnaire and COMQ-12 total score were highly significantly associated (p<0.01) with a higher score in the DASS-depression subscale score (Table 4).

**Table 4:** Univariate and multivariable logistic regression for DASS-depression subscale.

DASS-Depression	Univariate analysis		Multivariate analysis	
	B (95% CI)	p	B (95% CI)	p
**Sex**	0.23 (-0.4–0.85)	0.466		
**Age**	0.01 (0–0.03)	0.208		
**Type of COM**	0.19 (-0.82–0.44)	0.556		
**Duration of the disease**	0.13 (-0.44–0.18)	0.417		
**Education**	0.05 (-0.37–0.28)	0.774		
**In a marriage/ partnership**	0.05 (-0.73–0.63)	0.887		
**Better hearing ear PTA (dB)**	0.04 (0.02–06)	<0.001^a^	0.01 (-0.01–0.04)	0.283
**Worse hearing ear PTA (dB)**	0.03(0–0.05)	0.009^a^	0 (-0.03–0.02)	0.747
**COMQ-12 total**	0.07 (0.04–0.09)	<0.001^a^	0.68 (0.15–1.5)	0.109
Discharge from the ear	0.41 (0.2–0.61)	<0.001^a^	0.16 (-1.1–0.77)	0.732
‘‘Smelly ear”	0.15 (0.05–0.36)	<0.001^a^	1 (0.12–1.88)	0.026^a^
Hearing at home	0.52 (0.28–0.76)	<0.001^a^	0.02 (-1-1.1)	0.974
Hearing in groups or in noise	0.45 (0.2–0.7)	<0.001^a^	1.21 (0.16–2.27)	0.024^a^
Discomfort in ear	0.37 (0.18–0.56)	<0.001^a^	0.52 (0.33–1.37)	0.231
Dizziness	0.38 (0.16–0.6)	<0.001^a^	0.67 (0.22–1.56)	0.139
Tinnitus	0.45 (0.26–0.64)	<0.001^a^	0.37 (0.14–1.23)	0.392
Performing daily activities	0.46 (0.21–0.7)	<0.001^a^	0.53 (0.38–1.44)	0.255
Taking showers and baths	0.39 (0.21–0.57)	<0.001^a^	0.54 (0.32–1.41)	0.22
GP visits	0.35 (0.08–0.62)	<0.001^a^	0.98 (0–1.97)	0.051
Medication use	0.36 (0.1–0.62)	<0.001^a^	0.59 (0.37–1.55)	0.225
General impact	0.44 (0.21–0.66)	<0.001^a^	0.81 (0.1–1.71)	0.081

CI-confidence interval, DASS- Depression Anxiety Stress Scale, COM chronic otitis media, COMQ-12- Chronic otitis media questionnaire -12, PTA- Pure Tone Average, dB-decibels

^a^ p value <0.05

Age, PTA for the better ear and for the worse ear, and every item of the COMQ-12 questionnaire, including the COMQ-12 total score (p<0.05), were significantly associated with a higher anxiety DASS-anxiety subscale score (Table 5). The PTA for the better and for the worse ear, and every item of the COMQ-12 questionnaire, including the COMQ-12 total score (p<0.05), were significantly associated with a higher anxiety DASS-stress subscale score (Table 6).

**Table 5:** Univariate and multivariable logistic regression for DASS-anxiety subscale.

DASS-Anxiety	Univariate analysis		Multivariate analysis	
	B (95% CI)	p	B (95% CI)	p
**Sex**	0.55 (0.19–1.28)	0.143	0.0 (-0.02–0.015)	0.882
**Age**	0.02 (0–0.05)	0.043		
**Type of COM**	0.51 (0.23–1.26)	0.175		
**Duration of the disease**	0.06 (-0.3–0.42)	0.744		
**Education**	0.1 (-0.28–0.48)	0.596		
**In a marriage/ partnership**	0.48 (0.36–1.29)	0.267		
**Better hearing ear PTA (dB)**	0.1 (0.08–0.13)	<0.001^a^	0.02 (0–0.04)	0.043^a^
**Worse hearing ear PTA (dB)**	0.11 (0.09–0.13)	<0.001^a^	0.46 (0.3–0.07)	<0.001^a^
**COMQ-12 total**	0.19 (0.17–0.22)	<0.001^a^	0.14 (-0.57–0.86)	0.697
Discharge from the ear	1.16 (0.94–1.38)	<0.001^a^	0.4 (-0.4–1.21)	0.324
‘‘Smelly ear”	0.78 (0.55–1.01)	<0.001^a^	0.39 (0.37–1.16)	0.306
Hearing at home	1.51 (1.27–1.76)	<0.001^a^	0.26 (-0.67–1.2)	0.582
Hearing in groups or in noise	1.48 (1.23–1.74)	<0.001^a^	0.08 (-0.99–0.83)	0.867
Discomfort in the ear,	1.11 (0.91–1.3)	<0.001^a^	0.23 (-0.5–0.97)	0.531
Dizziness	1.02 (0.77–1.26)	<0.001^a^	0.03 (-0.73–0.8)	0.931
Tinnitus	0.87 (0.66–1.08)	<0.001^a^	0.17 (-0.57–0.91)	0.643
Performimg daily activities	0.92 (0.64–1.2)	<0.001^a^	0.27 (-1-0.51)	0.498
Taking showers and baths	0.93 (0.73–1.13)	<0.001^a^	0.6 (-0.81–0.68)	0.866
GP visits	1.26 (0.99–1.54)	<0.001^a^	0.03 (-0.88–0.82)	0.943
Medication use	1.08 (0.77-1-39)	<0.001^a^	0.31 (-1.14–0.52)	0.46
General impact	1.37 (1.14–1.59)	<0.001^a^	0.11 (-0.77–0.79)	0.979

CI-confidence interval, DASS- Depression Anxiety Stress Scale, COM chronic otitis media, COMQ-12- Chronic otitis media questionnaire -12, PTA- Pure Tone Average, dB-decibels

^a^ p value <0.05

**Table 6:** Univariate and multivariable logistic regression for DASS-stress subscale.

DASS-Stress	Univariate analysis		Multivariate analysis	
	B (95% CI)	p	B (95% CI)	p
**Sex**	0.52(0.25–1.29)	0.187		
**Age**	0.02 (0–0.04)	0.088		
**Type of COM**	0.2 (-0.98–0.58)	0.617		
**Duration of the disease**	0.02 (-0.39–0.36)	0.93		
**Education**	0.14 (-0.54–0,26)	0.494		
**In a marriage/ partnership**	0 (-0.87–0.88)	0.986		
**Better hearing ear PTA (dB)**	0.12 (0.09–0.14)	<0.001^a^	0.05 (0.03–0.08)	<0.001^a^
**Worse hearing ear PTA (dB)**	0.1 (0.08–0.12)	<0.001^a^	0.04 (0.01–0.06)	0.003^a^
**COMQ-12 total**	0.17 (0.14–0.2)	<0.001^a^	0.11(-0.73–0.95)	0.799
Discharge from the ear	1.05 (0.81–1.29)	<0.001^a^	0.18 (-0.77–1.13)	0.706
‘‘Smelly ear”	0.76 (0.51–1)	<0.001^a^	0.13 (-1-0.77)	0.771
Hearing at home	1.2 (0.92–1.48)	<0.001^a^	0.75 (0.35–1.85)	0.182
Hearing in groups or in noise	1.26(0.97–1.54)	<0.001^a^	0.45 (-0.62–1.53)	0.404
Discomfort in the ear	0.96 (0.74–1.18)	<0.001^a^	0.23 (-0.62–1.09)	0.603
Dizziness	0.87 (0.61–1.13)	<0.001^a^	0.16 (-1.06–0.75)	0.733
Tinnitus	0.78 (0.55–1.02)	<0.001^a^	0.2 (-0.68–1.07)	0.658
Performing daily activities	0.89 (0.58–1.19)	<0.001^a^	0.17 (-1.1–0.76)	0.721
Taking showers and baths	0.87 (0.65–1.08)	<0.001^a^	0.03 (-0.92–0.85)	0.942
GP visits	0.87 (0.54-1-21)	<0.001^a^	0.21 (-0.79-1-21)	0.682
Medication use	1.13 (0.83–1.44)	<0.001^a^	0.49 (-1.17–0.49)	0.327
General impact	1.35 (1.11–1.6)	<0.001^a^	0.42 (0.5–1.34)	0.368

CI-confidence interval, DASS- Depression Anxiety Stress Scale, COM chronic otitis media, COMQ-12- Chronic otitis media questionnaire -12, PTA- Pure Tone Average, dB-decibels

^a^ p value <0.05

Multivariate linear regression analysis was conducted to identify the predictors of DASS subscale scores in patients with COM. In establishing the predicting factors of DASS depression subscale, overall regression model was significant (F (15, 300) = 3.53, R2 = 0.15, p<0.01). Hearing problems when talking to people in groups or in noisy surroundings (1.21, 95% CI 0.16–2.27, p<0.05) and having a ‘‘smelly ear” (1, 95% CI 0.12–1.88, p<0.05) were significant positive predictors of a higher DASS-depression score. (Table 4) In establishing the predicting factors of DASS anxiety and stress subscales, overall regression models were significant (F (16, 299) = 22.89, R2 = 0.55, p<0.01 and F (15, 300) = 15.48, R2 = 0.44, p<0.01, respectively). Significant positive predictors of higher DASS-anxiety scores were PTA on the better (0.02, CI 95% 0.00–0.04, p<0.05) and worse hearing ears (0.46, CI 95% 0.3–0.07, p<0.01). Positive predictors of higher DASS-stress scores were PTA on the better (0.05, CI 95% 0.03–0.08, p<0.01) and worse hearing ear (0.04, CI 95% 0.01–0.06, p<0.01) (Tables 5 and 6).

## Discussion

Previous research indicates that there is a strong correlation between anxiety, depression and chronic disease, where up to 28% of individuals with a chronic condition have some sort of psycho-emotional disturbance [14–16]. In this study, a surprisingly high number of patients with COM (70.57%) met the criteria for anxiety measured with DASS-21. Almost half of patients (49.37%) reported mild to severe stress. Only 13.29% of patients had scores indicating depression disorder, which is similar to depression rates reported in the general population [17, 18]. Comparing to the results of the control group used in the study, levels of depression, anxiety and stress were significantly elevated in patents with COM.

Anxiety disorders seldom occur alone and are frequently associated with depression or other mental health problems [19]. Comorbidity with depression is estimated to range from 20% to 70% [20]. The majority of research found that patients tend to experience depression or anxiety after being diagnosed with a chronic disease or describe a cyclical relationship between the two [14]. In this study, DASS-21 depression, anxiety, and stress subscales’ scores each significantly positively correlated with established disease-specific symptom severity, health service and general life impact of the disease. Limitations imposed on a patient’s daily routines caused by the chronic disease could lead to frustration, sadness and distress [21–24]. Social isolation, less frequent contact with family and friends, everyday water precautions and the avoidance of certain situations are frequent in the lives of patients with chronic ear disease, especially in patients with hearing loss [6]. Anxiety and depression can be heightened in COM patients by uncertainty about the prognosis of the disease, fear of symptoms worsening and the impact of the disease on a patient’s future.

We hypothesized that other symptoms of COM could also be significant predictors of psychological distress; however, a poorer quality of life, more intense COM symptoms and demographic characteristics were not predictive of anxiety and stress levels. The only positive predictive factors of higher DASS-21 anxiety and stress scores were the higher values of PTA on the better and worse hearing ears. Hearing loss as an independent condition was already positively associated with anxiety disorders and anxiety symptoms [25, 26]. Previous studies demonstrated that COM patients with hearing loss reported physical role difficulties, poorer general health perception and social functioning when compared to those without hearing loss [27], but precise data on psychological well-being of these patients are still lacking. Weiss et al. indicated that there was a strong correlation between HRQOL and hearing thresholds in patients with cholesteatoma, but no connection with anxiety and stress levels was made [28]. A possible explanation for our results could be that hearing loss is a progressive and persistent symptom, while other symptoms of COM (such as ear discharge, ear discomfort, dizziness, tinnitus, performing daily activities, medication use, etc.) can be variable during the disease and depend on the present active infection of the middle ear space. In patients with a disposition to anxiety and stress, the level of disability caused by hearing loss can lead to a deepening feeling of inadequacy, insecurity and loss of control.

In our study, self-perceived hearing difficulty when talking to people in groups or in noisy surroundings was predictive for increased depression scores, which was partly expected. Depression following hearing loss has been consistently observed in different age groups. In the younger population, vulnerability to mental distress probably derives from barriers in communication and experiences related to stigma and discrimination, while in older groups, cognitive decline and social isolation combined with increased disability, morbidity and poorer health play a bigger role in the occurrence of depressive symptoms [29, 30]. Depression symptoms were observed both in patients with self-reported and measured hearing loss, and several large-scale studies have indicated that the severity of hearing loss is associated with increased depression risk [31, 32].

### Strength, limitations, and implications for practice

Successful management of the symptoms of COM is of primary concern for patients and the otosurgeon. The importance of depression screening in patients with COM has already been highlighted [7]. In this study, a high number of patients with COM reported symptoms of anxiety and stress that worsened with intensification of the disease symptoms. Hearing disability was the only positive predictor of increased levels of stress and anxiety in these patients. This made us question whether hearing loss is the single most disabling symptom of COM, while all the other COM symptoms are apparently more manageable by patients. It seems that hearing disability should be the focus of COM treatment to effectively help the patient and improve their mental health and quality of life. The importance of depression screening in patients with COM has already been highlighted [7]. The results of this study support the fact that psychological issues are to be expected and that they should be actively addressed when treating patients with COM in order to improve treatment results and the quality of life in these patients.

There are several limitations of this study. Despite a relatively large number of patients with COM with diverse demographic characteristics, this was a cross-sectional study conducted in a single medical center. Data were collected when patients were referred for a definitive treatment plan and the focus was only on previously untreated patients diagnosed with COM. The influence of further possible surgical or medical treatments on the level of psychological distress in patients was not examined and this should be reviewed in further studies.

## Conclusion

The study found a positive correlation between reported levels of anxiety, depression and stress, severity of COM symptoms and quality of life. However, in our patients with COM, only hearing disability was a significant positive predictor of anxiety and stress Positive predictors of depressive symptoms in COM patients were active drainage from the ear, more severe hearing problems noisy surroundings and tinnitus.
